# Recent developments in nucleic acid-based therapies for Parkinson’s disease: Current status, clinical potential, and future strategies

**DOI:** 10.3389/fphar.2022.986668

**Published:** 2022-10-20

**Authors:** Shivam Kumar Pandey, Rakesh Kumar Singh

**Affiliations:** Department of Pharmacology and Toxicology, National Institute of Pharmaceutical Education and Research, Raebareli, Uttar Pradesh, India

**Keywords:** neurodegeneration, α -synuclein, microRNA, short interfering RNA, short hairpin RNA, antisense oligonucleotides

## Abstract

Parkinson’s disease is the second most common progressive neurodegenerative disease diagnosed mainly based on clinical symptoms caused by loss of nigrostriatal dopaminergic neurons. Although currently available pharmacological therapies provide symptomatic relief, however, the disease continues to progress eventually leading to severe motor and cognitive decline and reduced quality of life. The hallmark pathology of Parkinson’s disease includes intraneuronal inclusions known as Lewy bodies and Lewy neurites, including fibrillar α-synuclein aggregates. These aggregates can progressively spread across synaptically connected brain regions leading to emergence of disease symptoms with time. The α-synuclein level is considered important in its fibrillization and aggregation. Nucleic acid therapeutics have recently been shown to be effective in treating various neurological diseases, raising the possibility of developing innovative molecular therapies for Parkinson’s disease. In this review, we have described the advancements in genetic dysregulations in Parkinson’s disease along with the disease-modifying strategies involved in genetic regulation with particular focus on downregulation of α-synuclein gene using various novel technologies, notably antisense oligonucleotides, microRNA, short interfering RNA, short hairpin RNAs, DNA aptamers, and gene therapy of vector-assisted delivery system-based therapeutics. In addition, the current status of preclinical and clinical development for nucleic acid-based therapies for Parkinson’s disease have also been discussed along with their limitations and opportunities.

## 1 Introduction

Parkinson’s Disease (PD) is the second most common neurological disorder, affecting 1% of the world population over the age of 60 (Chin-Chan et al., 2015; Aarsland et al., 2021). PD patients frequently experience non-motor symptoms such as REM (rapid eye movement) sleep behaviour disorder (RBD), olfactory impairment, and autonomic nervous system disorders such as constipation, bladder dysfunction, and orthostatic hypotension, in addition to the typical motor symptoms like resting tremor, rigidity, bradykinesia, gait disturbances, and postural instability (Titova et al., 2017; Kumar et al., 2022). The deterioration of dopamine pathways connecting substantia nigra pars compacta to the striatum is thought to be due to an abnormal build-up of α-synuclein in fibrillar form, resulting into dopamine shortage and impaired motor symptoms (Moon and Paek, 2015; Bloem et al., 2021). Although therapeutic administration of L-dopa, dopamine or dopamine receptor agonist precursor, can help with the improvement of motor symptoms of early-stage of the disease.

Furthermore, as neurodegeneration advances, the effect of drugs become shorter-lived or less effective, as with L-dopa and other dopamine agonists, which may cause significant motor and mental symptoms as well as wear-off and on-off occurrences (Sivanandy et al., 2021). Such problems associated with these therapies have rendered the researchers to look for alternative therapies including nucleic acid therapy for the patients with advanced PD. According to Braak’s theory, different symptoms of PD include pre-non-motor symptoms such as RBD, hyposmia, and digestive problems during various stages of the illness, at initial stage followed by cognitive and mental symptoms appearing later (Rietdijk et al., 2017; Schapira et al., 2017). According to this theory, the dorsal nucleus of the vagus nerve and the olfactory bulb, as well as possibly the periphery from the myenteric plexus to the lower brainstem, are the sites of atypical or severe accumulation/aggregation of α-synuclein, which is a major constituent of the hallmark pathologic lesions (including Lewy bodies and Lewy neurites) and whose mutations and multiplication are interconnected to dominantly inherited PD (Rietdijk et al., 2017). Hence targeting α-synuclein is considered as one of the critical approaches in slowing down the progression of neurodegenerative responses in PD. This article has emphasized on the prospects and approaches of nucleic acid therapy for treatment of PD by modifying α-synuclein expression through use of ASO (antisense oligonucleotides) and gene therapy, along with its current preclinical and clinical status.

## 2 Genetic dysregulations in Parkinson’s disease

The discovery of several genes associated with the pathophysiology of PD have offered relevant information about the molecular and cellular pathways involved in neurodegeneration in PD (Bandres-Ciga et al., 2020). In clinical settings, it may be difficult to distinguish between the patients with genetically determined PD from the individuals with sporadic PD. The autosomal dominant from of PD are included α-synuclein (SNCA), leucine-rich repeat kinase 2 (LRRK2), vacuolar protein sorting ortholog 35 (VPS35), eukaryotic translation initiation factor 4 gamma 1 (EIF4G1) (Bandres-Ciga et al., 2020). Furthermore, LRRK2 gene mutations are the most frequent cause of missense found in patients with familial as well as apparently idiopathic PD (Jeong and Lee, 2020). Even though there are 80 different forms of identified LRRK2 gene variants detected worldwide, but out of them approximately seven forms have been proven to be pathogenic (Ross et al., 2011; Jeong and Lee, 2020). The confirmed aberrant pathogenic mutation are Lrrk2, p.N1437H, p.R1441C/G/H, p.Y1699C, p.G2019S, p.R1628P, p.G2385R, and p.I2020T (Aasly et al., 2010; Kim and Jeon, 2014; Kestenbaum and Alcalay, 2017). On rare occasions, LB pathology is the dominant pathology in most cases of LRRK2-related PD (together, more rarely, with tau or TAR DNA-binding protein 43 (TDP-43 pathology) (Jeong and Lee, 2020; Rivero-Ríos et al., 2020). Additionally, the second leading cause of dominant PD is mutations in the SNCA gene (Bandres-Ciga et al., 2020). They comprise genome duplications and triplications, as well as rarer point mutations. The α-synuclein is produced by the SNCA gene and is present in Lewy bodies (LB) in the brainstem and other brain areas (Booms and Coetzee, 2021). Furthermore, VPS35 is a component of the endosomal–lysosomal trafficking retromer complex and is a vacuolar protein sorting 35 homologs (Sassone et al., 2021; Hong et al., 2022). Patients with the VPS35 variation mutation develop levodopa-responsive, late-onset PD, comparable to sporadic PD, but with an early onset age (Lin et al., 2019; Riboldi et al., 2022).

Moreover, the recessively inherited PD genes are the Parkin RBR E3 ubiquitin protein ligase 2 (PARK2), PTEN Induced Kinase 1 (PINK1), protein deglycase (DJ-1), ATPase Cation Transporting 13A2 (ATP13A2), Phospholipase A2 Group VI (PLA2G6), DNAJC6 (DnaJ Heat Shock Protein Family (Hsp40) Member C6) mutations are the monogenic causative factors in genetic regulations of PD (Kim et al., 2017; Aasly, 2020). Considering that autosomal recessive forms are PARK2 (parkin) variants are associated with the most frequent type of autosomal recessive PD, while PINK 1 and DJ-1 Lewy mutations also seem to be significantly less common (Niemann and Jankovic, 2019; Aasly, 2020). There are about a hundred different mutations in PARK2, comprising point mutations, duplications, and deletions that are homozygous or compound heterozygous (Jankovic et al., 2018; Selvaraj and Piramanayagam, 2019). The patients with sporadic, early-onset PD (Riboldi et al., 2022) have also been found to have PARK2 mutations (Kamienieva et al., 2021), though the importance of such result is still debated. The disease underlying PARK2-related PD, unlike autosomal dominant and idiopathic kinds of PD, does not usually present LB. Hence, motor changes are typical during illness. Moreover, mitogenesis, mitophagy, mitochondrial homeostasis, and mitochondrial transport are dependent on PARK2, PINK-1, and DJ-1 (Videira and Castro-Caldas, 2018; Nicoletti et al., 2021). The genetic variations in the ATP13A2 (ATPase type 13A2), PLA2G6 (phospholipase A2, group VI), and FBXO7 (F-box only protein 7) genes result in atypical parkinsonism, which are recessively inherited (Shen et al., 2018) Considering the fact that phenotypic traits genes have expanded new technologies for identifying the scope of both monogenic and risk-related PD (next-generation sequencing (NGS), genome-wide association studies (GWAS), the approaches that can be used to modulate these genes becomes quite important in PD (Grenn et al., 2020; Tan et al., 2021). The genetic dysregulations in PD causing autosomal dominant forms and autosomal recessive variant have been summarized in Table 1.

**TABLE 1:** Genetic dysregulations of different types of genes associated with PD.

Gene	Mutations	Genetic inheritance	Gene product	Phenotype	Pathology
Autosomal dominant PD					
LRRK2, leucine-rich repeated kinase 2 (PARK 8)	G2019S, N1437H, R1441C/G/H	AD (incomplete, age-dependent)	Lrrk2 (dardarin)	PD	ND, LB (rarely tau pathology)
	Y1699C, I2020T				
SNCA (PARK 1/4)	Triplication	AD (high)	α-synuclein	PD, MDD	ND, LB (point mutation), LB (multiplication)
	Duplication				
	A53T, A30P, H50Q, G51D, E46K				
EIF4G1	R1205H	AD	Eukaryotic translation initiation factor 4-gamma 1	PD	Regulation of activated PAK-2p34 by proteasome-mediated degradation
VPS35 (PARK 17)	D620N	AD (incomplete)	Vacuolar protein sorting 35 homolog	PD	axonal pathology
UCHL1 (PARK 5)	4P14	AD	Unknown	PD	Unknown
GIGYF2 (PARK 11)	2q37.1	AD	Unknown	PD	Unknown
Omi/HtrA2	2p12	AD	Unknown	PD	Unknown
Autosomal recessive PD					
PINK 1 (PARK6)	>40 different mutations	AR	PTEN-induced kinase 1	EO, PD	Unknown
ATP13A2 (PARK9)	Duplications	AR	Lysosomal P-type ATPase	EO PD, P-P	Unknown
	G877R, L1059, F182L, G504R				
PRKN (PARK2)	>100 different mutations	AR	Parkin, E3 protein ligase	EO PD	Pure ND (a single case with LB)
DJ-1 (PARK7)	>10 different mutations	AR	Daisuke-Junko-1	EO PD	Unknown
PLA2G6 (PARK14)	R741Q, R747W, Q452X, R635Q, R632W, D331Y	AR	phospholipase A2. Calcium-independent	EO PD, P-P	LB, tau pathology
DNAJC6	Unknown	AR	Neuronal-specific clathrin-uncoating co-chaperone auxilin	CO P-P	Unknown
FBXO7 (PARK 15)	R378G, R498X, T22M	AR	Only the F-box protein 7	PD, P-P	Unknown

DLB, dementia with lewy body; Dy, Dystonia; EOP, Early-onset parkinsonism; LB, lewy body; AD, autosomal dominant; AR, autosomal recessive; ND, nigral degeneration P, parkinsonism; O, occulomotor dysfunction; Psy, Psychiatric Syndrome; Pyramidal symbols; S, or spasm. Childhood Onset (CO), Early Onset (EO), and Late Onset (LO); atypical types of MDD, myoclonus, dementia, and dysautonomia P-P stands for parkinsonism-pyramidal syndrome. PD, traditional PD (levodopa-responsive parkinsonism).

## 3 Therapeutic approaches involved in genetic regulations in Parkinson’s disease

### 3.1 Downregulation of alpha-synuclein expression by therapeutics

**FIGURE 1:**
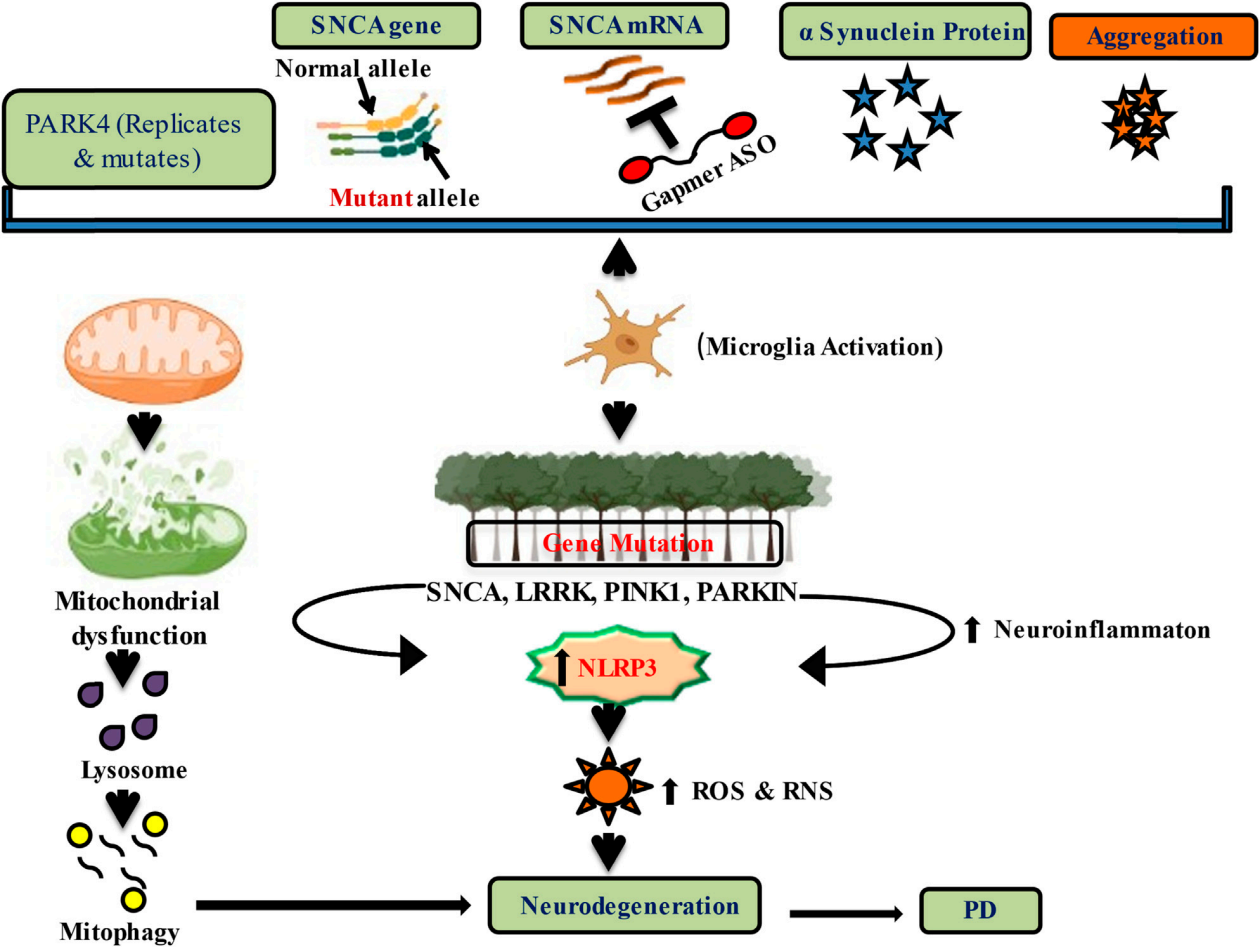
Downregulation of alpha-Synuclein expression and genetic regulation by nucleic acid therapeutics in PD.

Although abnormal or excessive levels of the protein α-synuclein can induce neurodegeneration in PD, lowering the expression of its genes LRRK2, PINK1, and VPS35 may help prevent progressive neurodegeneration (Figure 1) and hence may slow down further progression of the disease (Srinivasan et al., 2021).

Furthermore, the adeno-associated virus (AAV)-mediated generation of short hairpin RNA (shRNA) targeting SNCA in the substantia nigra has been shown to alleviate behavioural deficits in rats that have ectopically generated α-synuclein (Teil et al., 2020). Moreover, AAV-shRNA targeting SNCA reduces α-synuclein protein levels by around 35%, when delivered to the substantia nigra of wild-type rats, and protected from the neurotoxic mitochondrial rotenone (Zharikov et al., 2015). In addition to shRNA, and short interfering RNA (siRNA) have also been explored as potential suppressors of SNCA expression (Zharikov et al., 2015). The exosomes containing viral glycoprotein from rabies and siRNA directed against SNCA were delivered into the striatum and midbrain of mice, resulting in extensive siRNA distribution and a 50% reduction in SNCA mRNA and protein expression (Teil et al., 2020; Roshani et al., 2021). The infusion of siRNA and polyethyleneimine (PEI) against α-synuclein into the striatum resulted in a 65% reduction in SNCA mRNA expression and a 50% drop in α-synuclein protein (Singh and Sen, 2017).

### 3.2 Nucleic acid treatment to target other Parkinson’s disease genes

Nucleic acid treatment can also be used to target genetic alterations that cause dominantly inherited PD. LRRK2 mutations have been linked to the most common PD family type-8 (PARK8) (Jeong and Lee, 2020). It has been reported that shRNAs targeting mutations in LRRK2 elicit mutant allele-specific knockdown in human embryonic kidney (HEK 293FT) or (HEK 293) cells (de Yñigo-Mojado et al., 2011; Sibley and Wood, 2011). In addition, the animals receiving intracerebroventricular (ICV) injections or nigrostriatal injections of ASOs targeting LRRK2, which produced α-synuclein fibrils and α-synuclein inclusions resulted in lower levels of LRRK2 mRNA and protein expression (Zhao et al., 2017). The clinical use of ASOs to a variety of disorders is rapidly increasing due to their exceptional target specificity and long-lasting therapeutic benefits (Kulkarni et al., 2021). 

### 3.3 Approaches involved in Parkinson’s disease treatment using microRNA

MicroRNAs (miRNA) are non-coding RNAs that regulate gene expression after transcription by binding to the 3′-untranslated region of their target mRNAs (UTR). They are found to be present throughout the eukaryotic transcriptome and have a length of 20–25 nucleotides (O'Brien et al., 2018). However, miRNAs cleave/degrade mRNA or inhibit translation to regulate the expression of their target mRNA. In a healthy brain, miRNAs are crucial in controlling cell mRNA levels (Catalanotto et al., 2016). However, if abnormally produced, they can take on a pathogenic function, contributing to the underlying causes of PD. miRNAs are tiny RNA molecules that usually come from a “canonical” production route in the cell nucleus and degraded in the cytoplasm (O'Brien et al., 2018). There are two other sources of miRNAs: these molecules may be chemically produced as oligonucleotides that resemble the endogenous molecules, the miRNA mimics, for use in experimental studies, and they can also be created in cells *via* a “noncanonical” pathway. However, anti-miRNA compounds are used in the second method of miRNA-based therapies to cause a loss of function in the miRNA of interest (Qiu et al., 2015).

Furthermore, by injecting a complementary RNA sequence that binds to and inactivates target miRNAs, the intention is to inhibit these miRNAs in certain conditions where miRNAs are overexpressed (Catalanotto et al., 2016). Targeting miRNAs appears to be a possible therapeutic option for PD, even though there is no conventional medicine or active clinical studies ongoing so far (Li et al., 2020). The development of PD treatments through use of miRNAs that target and inhibit the production of α-synuclein seems to be one of the viable approaches.

In addition, while miRNA-7 loss causes α-synuclein build-up, which may be reversed with a miRNA replacement, this target also has other regulating genes involved in PD pathogenesis. Voltage dependent anion channel 1 (VDAC1) and Keap1 are downregulated by miRNA-7, which protects cells from reactive oxygen species. Additionally, it also inhibits the production of genes related to pro-inflammatory response (RelA and NLR family pyrin domain containing 3 (NRLP3), as well as cell glycolysis (RelA) (Lu et al., 2017). Considering that the disruption of mitochondrial activity is essential in the underlying causes of PD (Bose and Beal, 2016), targeting mitochondrial functions could also be important (Kalani et al., 2018). Additionally, VDAC1 also impact the deposition of mutant α-synuclein in the brainstem, striatum, and cortex, as well as the opening of mitochondrial permeability transition pore (mPTP) in A53T PD mice model (Alecu and Bennett, 2019). MiRNA-7 generated a convergent against α-synuclein deposition by targeting two different genes, VDAC1 and α-Syn. Furthermore, MiRNA-7 also protects against oxidative stress by inhibiting the cytoplasmic inhibitory protein Kelch-like ECH-associated protein 1 (Keap1) (Titze-de-Almeida and Titze-de-Almeida, 2018; Zhang et al., 2021). However, Keap1 is detected in physiological circumstances complexed with erythroid 2–related factor 2 (Nrf2) (Zhang et al., 2021), a nuclear protein that regulates antioxidant gene expression. Keap1 inhibits the migration of Nrf2 to the cell nucleus (Zhang et al., 2021). In this way, suppressing miRNA-7 over Keap1 will allow Nrf2 to move to the nucleus and activate genes that decrease oxidative stress. Overexpression of miRNA-7 increases glutathione levels in MPP^+^-damaged SH-SY5Y cells, resulting in a 50% decrease in hydroperoxides (Haque et al., 2020).

Furthermore, other miRNA approaches explored in PD are miR-124 and miRNA 155 (Zingale et al., 2021). In MPTP-challenged mouse model, miRNA-124 levels were shown to be lower, and administration of miR-124 to the right lateral ventricle protected tyrosine hydroxylase (TH) positive neurons from the toxin (Angelopoulou et al., 2019). In this context, mice injected with AAV2 α-synuclein had shown a significant increase in miRNA-155 levels in the substantia nigra, a well-known regulator of the acute and chronic inflammatory response (Zingale et al., 2021). In AAV2 α-synuclein–injected mice, however, ablation of the miR-155 gene inhibited stimulation of the microglia and attenuated TH-positive nigral neurons (Thome et al., 2016). Moreover, MiRNA-7, regulates both the level and synthesis of α-synuclein involved in neuroinflammation. Stereotactic injection of miRNA-7 into the mouse striatum has been shown to reduce neuroinflammation and offer neuroprotective advantages in MPTP-lesioned mice by targeting the 3′-UTR of NOD-like receptor protein 3 (NLRP3) mRNA (Zhou et al., 2016). The latter are involved in neuroinflammation and encode a protein that is a part of inflammasomes, which are common in microglial cells (Thome et al., 2016). Furthermore, this finding suggests that miRNA-7 inhibits NLRP3-mediated neuroinflammation, emphasizing role of miRNA-7 in various neuroprotective pathways and, its therapeutic potential for PD (Zhou et al., 2016).

The Drosha ribonuclease and Dicer enzymes successively degrade the long primary transcripts to make miRNAs (Treiber et al., 2019). Also, mice models have shown decreased dicer expression in the ventral midbrain in an age-elated fashion (Chmielarz et al., 2017). Furthermore, deletion of the Dicer gene in mice caused a gradual loss of dopaminergic (DA) neurons as well as substantial locomotor deficits (Airavaara et al., 2020). However, the latter finding indicates that the loss of mature miRNAs and the necessity of Dicer for DA neuron viability produced by Dicer gene deletion may play a role in the initiation and progression of PD (Chmielarz et al., 2017; Leggio et al., 2017). Interestingly, dicer deletion helps to increase the miRNA target gene in PD.

## 4 Therapeutic development of α-synuclein targeting siRNA

### 4.1 Conjugates of GalNAc-siRNA

siRANA approach offers much promise for a variety of indications from cancer to pandemic virus outbreaks to neurodegenerative diseases including PD. A trimer of N-acetyl galactosamine (GalNAc) is the prototype siRNA conjugate, and it preferentially binds to the asialoglycoprotein receptor (ASGPR), which is mainly expressed in PD (Springer and Dowdy, 2018). Moreover, GalNAc-siRNA conjugates have been comprehensively explored in preclinical rodent and non-human primate (NHP) models. They are now undergoing clinical studies supported by three different biotech firms (Andersson et al., 2018). GalNAc conjugation effectively increases siRNA target organ accumulation, and make cellular absorption easier.

Consequently, siRNA must be chemically treated to maintain stability in the circulation after parenteral injection without a protective delivery vector (Springer and Dowdy, 2018). In addition, these treatments are made up of siRNA conjugated to a triantennary GalNAc moiety that targets the ASGPR to suppress disease-causing genes in PD (Adachi et al., 2021).

### 4.2 Short hairpin RNA

In order to introduce shRNAs into cells, and transform them into siRNAs, and then use the RNAi machinery to effect targeted gene suppression, bacterial or viral vectors are required (Tam et al., 2017). Because the host cells can continually generate shRNAs, they offer several benefits than siRNA, including more prolonged effects, reduced dose requirements, and fewer impacts that are both particular and general off-target. Moreover, there are also drawbacks of utilising viral vectors, such as immunogenicity and the potential to generate mutations, which make viral vectors potentially dangerous (Lin et al., 2017). In normal rats, adeno-associated virus-mediated administration of shRNAs caused a knockdown of α-synuclein of 35% without compromising major motor function or triggering dopaminergic neuron deterioration.

Furthermore, α-synuclein knockdown using shRNAs proved to be neuroprotective in a PD rat model, reducing dopaminergic neuron loss and slowing the onset of motor impairments (Zharikov et al., 2015). In a PD mouse model, shRNAs were utilised to knock down polypyrimidine tract binding protein 1 (PTBP1), and transformed astrocytes to dopaminergic neurons, correcting motor impairments and replacing dopaminergic cell loss (Qian et al., 2020). Further preclinical research is required open up potential new therapeutic avenues for PD and other neurodegenerative diseases.

Although one of the published studies has reported that the shRNAs decreased the expression of α-synuclein and attenuated dopamine neuron degeneration in rat PD model induced with 1-methyl-4-phenyl 1,2,3,6-tetrahydropyridine (MPTP) (Liu et al., 2014; Zhang et al., 2016), however, other techniques, such as decreasing inflammation *via* class II transactivator or caspase-1 inhibition by shRNA, have also been tried instead of directly targeting SNCA (Li et al., 2020). In addition, shRNAs targeting nuclear receptor related 1 (Nurr1) or Src homology region 2 (SH2)-containing protein tyrosine phosphatase 2 (Shp2) were developed as a viable method for treating levodopa-induced dyskinesias (Sellnow et al., 2015). However, because Nurr1 performs numerous roles, including protection of dopaminergic neurons against neurotoxins and regulating neuroinflammation, hence further studies are needed to validate Nurr1-based PD therapies (Kandil et al., 2018; Paliga et al., 2019).

## 5 Parkinson’s disease treatment using novel DNA aptamers

Small single-stranded DNA (ssDNA) or RNA molecules are known as aptamers with excellent affinity and specificity for a wide range of target proteins (Navien et al., 2021). They are referred to as “chemical antibodies” and are frequently used to replace chemical antibodies (Zhou and Rossi, 2017). Aptamers differ from traditional antibodies because they have their own characteristics (Byun, 2021). Moreover, aptamers used as an nucleic acid therapy, for instance, are neither immunogenic nor toxic compounds since the human immune system seldom recognizes them as foreign substances; they are more thermally stable and can maintain their structures over several cycles of denaturation and renaturation, and they are conveniently labelled and adjusted (Zhou and Rossi, 2017; Byun, 2021). Furthermore, the aptamers can distinguish between various conformations of the same relevant target protein. A DNA aptamer named A1 was chosen for neurological illnesses because it has a strong affinity and selectivity for the b-site amyloid precursor protein cleaving enzyme 1 (BACE1), which decreases BACE1 activity and lowers beta-amyloid formation (Ab) (Zheng et al., 2018).

In addition, a DNA aptamer that can precisely detect and attach to amyloid beta oligomer was developed (Kim et al., 2020). Although various DNA aptamers against α-synuclein have been tested in PD, however, these aptamers have limited affinity and specificity due to selection restrictions. Yet, there are limited evidence reported using aptamers to control α-synuclein toxicity. The discovery of the first DNA aptamer that binds to α-synuclein sparked interest in study of such molecules in PD research, including pathogenesis studies and the development of diagnostics and therapies (Zheng et al., 2018). Based on additional findings that antibodies targeting the C-terminus of α-synuclein reduced the extent of oligomerization and mitigated the loss of nigral dopaminergic neurons, two aptamers selected from 11, 019 sequences were found to bind to α-synuclein with high affinity (Teil et al., 2020). Moreover, aptamers have also been used to monitor dopamine levels, with “aptasensors” like the ultrasensitive and selective voltametric aptasensor and the gold nanoparticle improved surface plasmon resonance aptasensor reportedly being utilized to detect dopamine levels as low as 200 fantomolar (Cao and McDermott, 2018). The development of aptasensors as tools for monitoring disease progression in clinical trials and performing clinical diagnosis is aided by their higher specificity and sensitivity in detecting dopamine (Li et al., 2019). Although aptamer treatments have also shown several drawbacks, including rapid clearance, metabolic instability, and inadequate translational *in vivo* correlation, however, they are supposed to have enormous potential as innovative medicines with enhanced neurobiological activities (Zhou and Rossi, 2017).

## 6 Vector-assisted delivery systems

### 6.1 Adeno-associated virus vectors for Parkinson’s disease gene therapy

Adeno-associated viruses (AAVs) are single-stranded DNA viruses that are tiny and non-enveloped. They belong to the *Parvoviridae* family (Li et al., 2019; Fajardo-Serrano et al., 2021). Despite their small size, they are the most promising vehicle for CNS gene therapy because they are clinically safe and efficient in transduction of proliferating and quiescent cells, and they can induce long-term transgene expression (Fajardo-Serrano et al., 2021). AAV-based vectors have been used nearly entirely in PD gene therapy clinical studies. AAV serotypes are a primary factor of biodistribution, tissue tropism, and sensitivity to neutralising antibodies developed *in vivo*, all of which are essential properties of effective AAV-based gene therapy (Choudhury et al., 2016; Fajardo-Serrano et al., 2021). To build a robust and predictable gene therapy technique, it is needed to figure out how distinct serotypes transport gene cargos may reach to their intended locations for vector delivery. In humans and non-human primates, more than a hundred AAV variants with 12 serotypes (AAV1e12) have been found (Hammond et al., 2017). Furthermore, AAV2 has also been employed in multiple clinical trials. It is now regarded as a suitable vector for gene treatment of neurodegenerative illnesses such as PD due to its relative safety profile and persistent expression in neurons.

#### 6.1.1 Adeno-associated virus type 2 vector-glutamic acid decarboxylase gene therapy

PD is characterised by the death of dopaminergic neurons in the SN and numerous brainstem, limbic, and midbrain neurons, resulting in changes in the activity of movement-controlling brain networks (Khan et al., 2020). It is categorized by dysregulations of interacting inhibitory and excitatory pathways leading to the difficulties in movements, muscle stiffness and tremors (Bouabid et al., 2016; Murueta-Goyena et al., 2019). Most patients benefit from pharmacological facilitation of dopaminergic neurotransmission initially however, those with severe PD are more likely to experience drug-related issues such as impaired voluntary movement and motor irregularities (Bouabid et al., 2016). Direct therapies that increase dopaminergic neurotransmission after the onset of these difficulties may have negative consequences and aggravate dyskinesia (Murueta-Goyena et al., 2019).

Furthermore, increased subthalamic nucleus (STN) activity is primarily mediated by a loss in GABAergic input from the globus pallidus in individuals with PD (Murueta-Goyena et al., 2019; Muñoz et al., 2020). Electrical stimulation, STN lesioning, and gamma-aminobutyric acid (GABA) infusion have all been demonstrated to reduce STN activity in clinical investigations. In animal models of PD, glutamic acid decarboxylase (GAD) gene transfer and other approaches that modify GABA synthesis in the subthalamic nucleus enhance basal ganglia function (Muñoz et al., 2020). Gene therapy might be a viable treatment option, which involves inserting the glutamic acid decarboxylase gene (GAD) into the subthalamic nucleus (Shalaby and El-Agnaf, 2022). GAD is the rate-limiting enzyme for GABA synthesis, and PD affects the function of both GABA efferents to the subthalamic nucleus and their targets within the basal ganglia circuitry (Stoddard-Bennett and Reijo Pera, 2019; Muñoz et al., 2020). An infusion of the GABAergic agonist, muscimol into the subthalamic nucleus during deep brain stimulation (DBS) surgery in patients with PD (Heiss et al., 2019) suppressed its neuronal ring rates and temporarily improved PD symptoms, suggesting enhanced GABA transmission within the subthalamic nucleus could be beneficial in PD. Gene transfer of GAD had similar outcomes in animal models of PD (Niethammer et al., 2017; Heiss et al., 2019). This method delivers GAD to the subthalamic nucleus using an adeno-associated viral vector (AAV2) to restore local GABA transmission and normalise output from the nucleus (by adding an inhibitory GABA Outflow, thereby reducing excessive excitatory glutamate output to critical targets such as the globus pallidus interna and the substantia nigra reticulata) (Niethammer et al., 2017). AAV2-GAD injected unilaterally into the subthalamic nucleus was found to be safe and linked with improvements in parkinsonism in an open-label clinical experiment (Allen and Feigin, 2014; Merola et al., 2021). In individuals with advanced PD, a variety of clinical trials were conducted to test the effects of bilateral AAV2-GAD administration into subthalamic nucleus against bilateral sham surgery (Niethammer et al., 2017).

#### 6.1.2 Treatment with Adeno-associated virus type 2 vector-GDNF

Glial cell line-derived neurotrophic factor (GDNF) is a substance that may preserve and enhance dopamine-producing brain cells (Manfredsson et al., 2020). PD affects dopamine neurotransmitter production in the brain function (Bloem et al., 2021). Furthermore, AAV2-GDNF, the gene used for the treatment of PD, may aid in producing GDNF, which protects injured brain cells (Cortés et al., 2017). GDNF, a member of the transforming growth factor β (TGFβ) family, is a potent neurotrophic factor required for dopaminergic neuron outgrowth and survival (Mahato and Sidorova, 2020), making it a promising candidate for protecting existing neurons from environmental insults and restoring function in the affected neurons. However, because GDNF is rapidly destroyed in the human body and does not cross the blood-brain barrier well (Deierborg et al., 2008), the only effective method of its administration is its instillation into appropriate brain regions (Barker et al., 2020). In early clinical trials, bolus injections of GDNF into the lateral ventricle resulted in little clinical benefit, significant side effects, and the formation of antibodies against GDNF in some patients (Barker et al., 2020). When injected into the striatum, AAV was successfully tested in producing continuous expression of GDNF in a rat model of PD through direct injection into either striatum or substantia nigra (Negrini et al., 2022). Similarly, LV-GDNF was shown to be effective in the reversal of PD symptoms in rhesus monkeys (Axelsen and Woldbye, 2018). The AAV2-GDNF vector was delivered into the putamen of two distinct rhesus monkey models using a convection-enhanced technique. They describe clinical improvement in MPTP-lesioned PD monkeys, as well as indications of functional recovery in the nigrostriatal pathway, without any major adverse effects and persistent GDNF expression over a period of 12 months (Albert et al., 2017; Grondin et al., 2019). AAV2-GDNF was also given to elderly monkeys with a naturally occurring reduction of motor activity, resulting in increased dopamine analogue absorption and improved locomotor activity (Grondin et al., 2019). When high-dose vector particles were delivered, the density of dopaminergic terminals in the putamen increased, as did the number of non-pigmented neurons in the SN, with no significant adverse effect (Albert et al., 2017). On the other hand, the convection improved delivery of AAV2-GDNF in phase-1 clinical study, which is evaluating the efficacy and safety of AAV2-GDNF genetic transfer to treat advanced PD (McFarthing et al., 2019; Fiandaca et al., 2020; Van Laar et al., 2021).

#### 6.1.3 Gene therapy using Adeno-associated virus type 2 vector-AADC

One of the primary concerns in PD therapy is that the sole medicine available, levodopa becomes less effective along with the progression of the disease. The efficacy of levodopa has been found to decrease with time, when AADC levels fall (Billings et al., 2019). This has led to efforts to use AAV-mediated gene therapy to boost AADC expression. Delivering AADC to the brain might increase dopamine function and restore therapeutic efficacy of levodopa (Bankiewicz et al., 2006; Blits and Petry, 2017). It has been shown in a clinical trial that AAV vector encoding AADC, delivered to the putamen by MRI-guided convection-enhanced diffusion (CED) delivery (San Sebastian et al., 2012; Sudhakar and Richardson, 2019). The patients also had positron emission tomography (PET) scans with AADC-specific tracer [18F] fluoro-L-m-tyrosine (FMT) 1–10 days before surgery and 1and 6 months following surgery, and yearly for up to 5 years (Sudhakar and Richardson, 2019). Furthermore, PET imaging demonstrated a considerable increase in AADC expression throughout first 6 months. This outcome was accompanied by a favourable safety profile and tentative clinical benefit indications (Blits and Petry, 2017). Long-term follow-up of these patients revealed that the rise in AADC was sustained throughout the study period, suggesting that the expression remained permanently elevated (Axelsen and Woldbye, 2018). In an open-label phase 1 experiment in PD patients, the locomotor score of the universal PD rating scale (UPDRS) in the off-L-dopa condition improved by 36% after the introduction of the AADC-expressing AAV type 2 vector (AAV2-AADC) into the putamen bilaterally (Axelsen and Woldbye, 2018). Although this clinical trial was not designed to track effectiveness, temporal assessments of UPDRS (Unified PD Rating Scale) scores in the ON and OFF modes revealed a substantial improvement in all patients in the first 12 months. Another study have investigated the safety, tolerability, and possible effectiveness of AAV vector-mediated gene delivery of AADC into the putamen of six individuals with PD (Albert et al., 2017). They found that intra-putaminal in a non-human monkey model of advanced PD, gene transfer of dopamine-synthesizing enzymes, particularly AADC, resulted in high transgene expression and restoration of putaminal DA amounts. (Wang et al., 2002). Furthermore, the treatment significantly improved hand dexterity, tremor, bradykinesia, and muscle stiffness, effectively restoring motor functioning. Up to 15 years following therapy, long-term healing and transgenic expression were seen (Sehara et al., 2017). There was no evidence of cytotoxicity or Lewy body disease in the transduced neurons, which were widely scattered throughout the putamen (Sehara et al., 2017). During simultaneous peripheral levodopa treatment, microdialysis revealed higher extracellular DA in the overexpressed transduced putamen. These data illustrate the efficacy and safety of AADC gene therapy over the long term intraputamen delivery in PD (Sehara et al., 2017; Axelsen and Woldbye, 2018).

**FIGURE 2:**
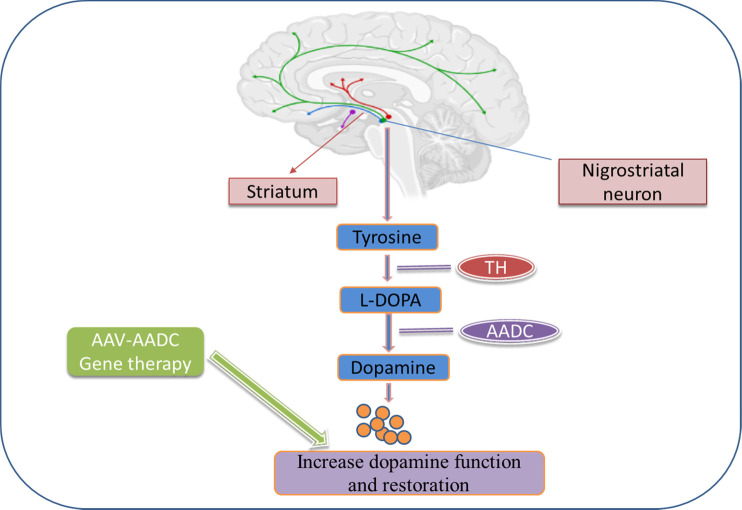
Dopamine biosynthesis in the striatum and gene therapy approaches to increase dopamine production and restoration in PD. In the striatum, tyrosine is converted to L-dopa by tyrosine hydroxylase (TH) and further converted into dopamine by aromatic L-amino acid decarboxylase (AADC). Gene therapy for PD introduces these enzymes into the striatum using viral vectors.

Furthermore, three specific phase 1 studies, two in Unite States and one in Japan have evaluated putamen-specific AADC-gene therapy in 31 patients, including its safety, pharmacodynamics, and preliminary effectiveness, two in the United States and one in Japan (Christine et al., 2019). UPDRS and PET using an AADC tracer were the essential measurements. At 6 months after the injection, the OFF-state’s motor score had improved by 46%, but PET levels had grown by more than 50% and remained high for 96 weeks, indicating permanent transduction of the putamen to express AADC (Blits and Petry, 2017). According to their research, these findings merit further investigation in a randomised phase II study. Thus, AAV2-AADC gene therapy could be potential vector therapy for PD to alter the disease progression (Figure 2).

## 7 Therapeutic potential of these approaches

Nucleic acid therapies have shown considerable promise in treating various neurological illnesses such as PD, and they provide numerous key benefits, including excellent target specificity, stability, minimal toxicity, and immunogenicity (Jurgielewicz et al., 2021). The simplicity and predictability of chemical production of nucleic acid therapies, as well as the quick drug development mechanism and characteristic pharmacokinetic parallels, are all significant benefits (Yamada, 2021). Nucleic acid therapies offer much potential in PD, and several studies have already demonstrated that they can change the expression of essential PD proteins, including α-synuclein and its isoforms (Uehara et al., 2019; Zhao et al., 2021). Although environmental factors such as pesticides or metals may contribute to α-synuclein aggregation, and the gastrointestinal microbiome may influence PD drug metabolism or clinical phenotypes of the disease (Simon et al., 2020), however, nucleic-acid based therapies promise potential novel routes and genetic target for modification of the disease pathology (Kulkarni et al., 2021).

## 8 Current status: Preclinical and clinical data

The dopaminergic pathways have recently been restored using various gene therapy techniques (Blits and Petry, 2017). Several preclinical research have investigated striatal viral transduction and expression of AAV-GDNF, AAV-AADC, AADC alone, and AAV-GAD in NHP models of PD (Kirik et al., 2017; Axelsen and Woldbye, 2018). These investigations showed improved motor behaviour, safe gene transfer, strong expression, and restoration of DA signalling in PD like symptoms associated with primates and NHPs (Kirik et al., 2017).

Several pivotal clinical trials have evaluated dose escalation, safety, and efficacy of gene therapy in PD patients including safety study of AADC gene therapy, genetic convection-enhanced delivery/AAV2-GDNF (Björklund et al., 2000; Bäckman et al., 2006; McFarthing et al., 2019), AAV2-NTN (Marks et al., 2008), AAV-hAADC-2 (Christine et al., 2019; McFarthing et al., 2019; Christine et al., 2022), and rAAV-GAD (McFarthing et al., 2019); but none are currently accessible to PD patients. The genetic therapies implicated in preclinical and clinical research in PD have been listed in Table 2 and Table 3.

**TABLE 2:** Preclinical data of gene therapy for Parkinson’s Disease.

Vector gene delivery	Method	Subjects	Results	References
AAV-GDNF	Unilateral MRI-guided convection-enhanced delivery of either low titer or high titer AAV5-GDNF vector to putamen	Macaque monkeys (*n* = 4)	Higher GDNF expression with higher doses in both SN and striatum	Emborg et al. (2014)
	Bilateral AAV2-GDNF delivery to putamen after lesion	Unilaterally MPTP-treated macaques (*n* = 15)	Robust improvement of motor behaviour at 24 months. Effect proportional to the severity of the lesion. 3-fold increase in striatal dopamine	Kells et al. (2010)
	Unilateral injection of AAV2-GDNF in putamen after MPTP-lesioning	Unilateral MPTP-lesion Rhesus monkeys	Bilaterally increased striatal metabolism. 60 ± 6% decrease in CRS scores	Eberling et al. (2009)
	Bilateral CED of AAV2-GDNF in either putamen with high- or low titer or SN.	Macaques aged >20 years (*n* = 17)	No detectable immunological response to therapy after 6 months. SN group showed significant weight loss (–19.4%) not attributable to therapy	Su et al. (2009)
	Bilateral CED of AAV2-GDNF in putamen 4 months after MPTP-lesion	Unilaterally MPTP-treated macaques (*n* = 11)	No detectable immunological response. Bilaterally enlarged TH + fibers in the striatum	Su et al. (2009)
	Unilateral injection of AAV2-GDNF in the striatum and SN 4 weeks before	Marmoset monkeys (*n* = 11) with unilateral 6-OHDA mediated nigral forebrain bundle lesion	19% survival of nigral dopaminergic cells. Uncertain mitigation of parkinsonian behavior	Eslamboli et al. (2003)
	Unilateral injection of LV-GDNF into the caudate nucleus, putamen and SN	Macaques aged between 24 and 27 years (*n* = 4)	Unilateral >800% increase in TH-positive cells in the striatum	Palfi et al. (2002)
	Bilateral injection of AAV-GDNF a vector in the caudate nucleus. Euthanasia 1 week after operation	St. Kitts green monkeys (*n* = 4)	First proof that humane GDNF can be expressed in primates utilizing an AAV-vector. Increased GDNF amount in striatum	Kozlowski et al. (2001)
	Unilateral injection of LV-GDNF into the caudate nucleus, putamen and SN.	Aged non-lesioned macaques approx. 25-years-old (*n* = 8)	Robust antero- and retrograde distribution of GDNF-vector. The strong trend towards unilateral improved 18 F-DOPA uptake. 85% increase in TH-positive neurons on the treated side	Kordower et al. (2000)
	Unilateral MRI-guided injection of LV-GDNF in either putamen, SN or caudate nucleus after MPTP lesion	Unilaterally MPTP-treated young macaques (*n* = 12)	44% increase in TH-immunoreactive cells in striatum compared to controls. >300% increase in FD-uptake in left striatum. Rescue of motor behaviour in all groups	Kordower et al. (2000)
AAV- AADC	8-year follow-up on the study	MPTP-lesioned macaque monkeys (*n* = 2)	No decrease in FMT signal and continued robust AADC expression	Hadaczek et al. (2010)
	Unilateral AAV-2 induced AADC expression in striatum	MPTP-lesioned macaque monkeys (*n* = 8)	PET-verified increase in AADC activity after 2 years. Consistently higher L-DOPA sensitivity in the treatment groups	Bankiewicz et al. (2006)
	Dose-ranging study with bilateral putaminal injection of AAV2-AADC in escalating doses	MPTP-treated macaque monkeys (*n* = 12)	A minimum dose is needed to increase L-DOPA response and FMT activity in the striatum	Forsayeth et al. (2006)
	Bilateral convection-enhanced delivery AAV induced AADC expression in nu. caudatus, putamen and globus pallidus	MPTP-treated macaque monkeys (*n* = 4)	Verification of strong striatal AADC expression after 3 years	Daadi et al. (2006)
	Bilateral convection-enhanced delivery AAV induced AADC expression in nu. caudatus, putamen and globus pallidus	MPTP-treated rhesus monkeys (*n* = 4)	a higher increase in gene expression using CED than conventional injection. Strong AADC expression in the striatum	Bankiewicz et al. (2000)
AAV- GAD	Unilateral AAV-mediated GAD expression in STN after MPTP-lesion	MPTP-treated rhesus monkeys (*n* = 13)	Well tolerated. No overall decrease in morbidity. Sub-analysis with decreased bradykinesia −16%, gross motor skills (–26%) and tremors (–36%). Unilaterally decreased FDG-metabolism	Emborg et al. (2007)
	Unilateral recombinant adeno-associated virus (rAAV) to transduce neurons in the STN after 6-OHDA lesion	6-OHDA -treated rats (n = 13)	GAD gene transfer into glutamatergic excitatory neurons, leading to an inhibitory bias with altered network activity and a neuroprotective phenotype holds potential for the treatment of PD	Luo et al. (2002)

**TABLE 3:** Clinical trial data of gene therapy for Parkinson’s Disease.

Study title	Status	Study results		Conditions	Interventions	ClinicalTrials.gov identifier
Safety Study of AADC Gene Therapy (VY-AADC01) for Parkinson’s Disease	Completed	No Results Available	Parkinson’s Disease		Biological: VY-AADC01	NCT01973543
Safety Study of Subthalamic Nucleus Gene Therapy for Parkinson’s Disease	Completed	No Results Available	Parkinson’s Disease		Genetic: Surgical infusion of AAV-GAD into the subthalamic nucleus	NCT00195143
AAV2-GDNF for Advanced Parkinson’s disease	Completed	No Results Available	Parkinson’s Disease		Genetic: Convection enhanced delivery/AAV2-GDNF	NCT01621581
Safety of CERE-120 (AAV2-NTN) in Subjects with Idiopathic Parkinson’s Disease	Completed	No Results Available	Parkinson’s Disease		Genetic: CERE-120: AAV2-NTN	NCT00252850
A Study of AAV-hAADC-2 in Subjects with Parkinson’s Disease	Completed	No Results Available	Parkinson’s Disease		Genetic: AAV-hAADC-2	NCT00229736
GALIG Gene Expression in Parkinson’s Disease	Completed	No Results Available	Parkinson’s Disease		Other: Blood sampling	NCT02923297
GDNF Gene Therapy for Parkinson’s Disease	Recruiting	No Results Available	Parkinson’s Disease		Biological: AAV2-GDNF	NCT04167540
Safety and Efficacy Study of VY-AADC01 for Advanced Parkinson’s Disease	Active, not recruiting	No Results Available	Parkinson’s Disease		Drug: VY-AADC01	NCT03065192
Safety and Efficacy Study of VY-AADC01 for Advanced Parkinson’s Disease	Active, not recruiting	No Results Available	Parkinson’s Disease		Drug: VY-AADC01	NCT03065192
AADC Gene Therapy for Parkinson’s Disease	Terminated	No Results Available	Parkinson’s Disease		Genetic: Cohort1 and Genetic: Cohort2	NCT02418598
Long Term Follow-Up Study for rAAV-GAD Treated Subjects	Terminated	No Results Available	Parkinson’s Disease		Biological: rAAV-GAD	NCT01301573
Study of OXB-102 (AXO-Lenti-PD) in Patients with Bilateral, Idiopathic Parkinson’s Disease	Terminated	No Results Available	Parkinson’s Disease		Drug: OXB-102 Other: Imitation Surgical Procedure (ISP)	NCT03720418
miRNA therapy for Parkinson’s Disease						
miRNAs Profiling in Parkinson’s Disease	Active, not recruiting	No Results Available	Parkinson’s Disease		Genetic: biomarker identification in Parkinson’s disease	NCT03466723

## 9 Conclusion and future perspectives

This research has assessed and may provide improvements in our understanding of complicated molecular and genetic nucleic acid therapies for PD, as well as the proteins and genes involved in familial and sporadic forms of the neurodegenerative disorder. It also highlights the need for subtyping driven by biomarkers of PD as a platform for developing novel therapeutic options, including nucleic acid-based therapies, employing a nucleic acid medicine strategy. Such a strategy is a sensible and straightforward approach to tackling the heterogeneity concern in PD and finding molecular hereditary pathways that are more unique to various diseases, as well as potential targets for innovative disease-modifying medicines. As a result, the promise of nucleic acid-based treatments in PD are large and diverse, including a wide range of approaches and targets. While none are currently accessible to PD patients, the potential is evident in the future, and recent advances in other neurologic disorders provide a firm hope.
